# Supplementary material for: Recombinant Plasminogen Activator of the Sandworm (Perinereis aibuhitensis) Expression in Escherichia coli
Source: Bioengineering (Basel). 2024 Oct 15;11(10):1030. doi: 10.3390/bioengineering11101030 (PMC11504054; doi:10.3390/bioengineering11101030)
Supplement: Supplementary file 1 [file bioengineering-11-01030-s001.zip › Supplementary Materials S1-CL4328 COA.pdf]

Certificate of Analysis

Project ID: C907J671G0-2

Construct Information:

Gene Name: pET28a-pwPlasmin_pET-28a(+)

Clone ID: KK017599 **Gene Length:** 797 bp

Cloning Vector: pET-28a(+) **Cloning Strategy:** NcoI/XhoI

Growth in Bacteria:

Plasmid resistance: Kanamycin

Suggested competent cell: TOP10

Growth Temperature: 37 °C

QC Items	Specifications	Results	
**Appearance**	Colorless, clear, free of precipitate or foreign particles	Pass	Clear, colorless, no visible particulates
**Sequence Accuracy**	Sequencing verification match the order requirements	Pass	Matched
**Restriction Digests**	Expected size bands detected in agarose gel electrophoresis.	Pass	Matched<br>Shown in attachment 1
**A260/280**	1.8~2.0	Pass	1.94
**Quantity**	Miniprep: 4 µg	Pass	≥ 4 µg
**Additional Tests**	N/A	N/A	

Default	Plasmid Storing at	Bacstab Storing at	Glycerol Stock Storing at
Lyophilized	-20°C	4°C	-80°C

Certified by: Jing Yu **Date:** 11/24/2023

江苏省南京市江宁科学园雍熙路28号

电话：400-025-8686 025-58897288-5820 传真：025-58897288-5815 电子邮箱：order@genscript.com.cn 网址：www.genscript.com.cn

Attachment 1

Plasmid Construct Map

The gene was cloned in pET-28a(+) by NcoI/XhoI .

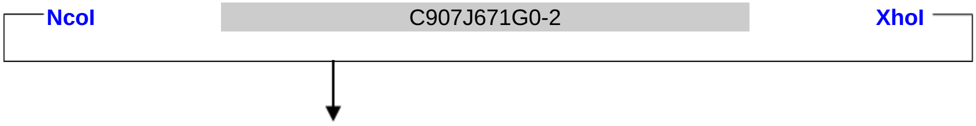

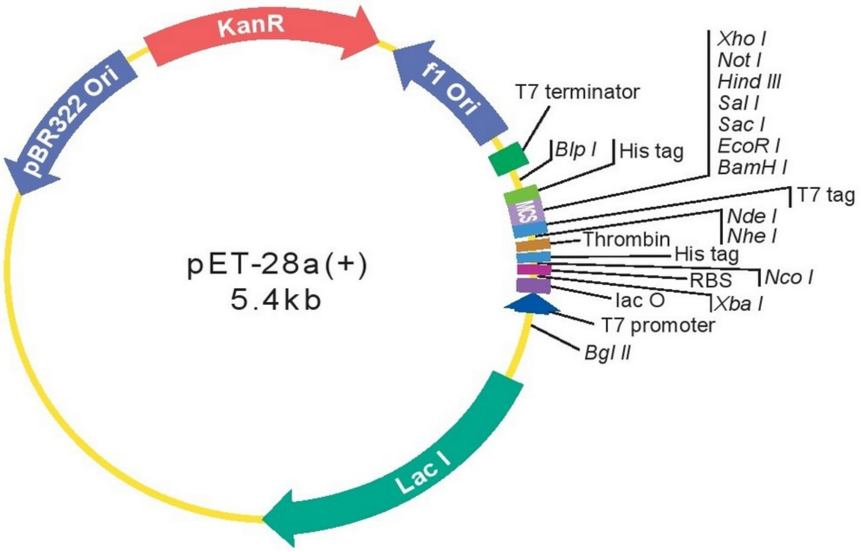

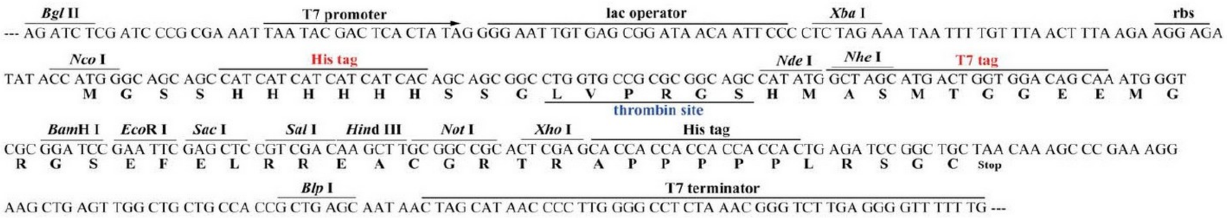

Notes: This map is based on a commercial vector. Some of the clone sites may be lost after cloning.

Enzyme Digestion

Lane M: KB Ladder
Lane 1:C907J671G0-2 plasmid
Lane 2:C907J671G0-2 plasmid digested
by EcoRI and XhoI

Restriction digest of plasmid DNA:
About 200-1000 ng of plasmid was digested at 37°C for 30-60 minutes and analyzed on 1% Agarose Gel

1 2 M

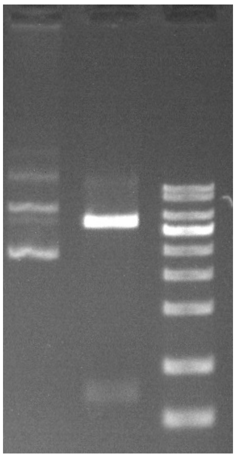

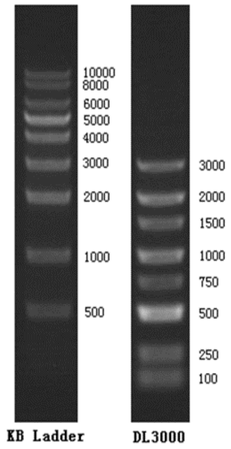

江苏省南京市江宁科学园雍熙路28号
电话：400-025-8686 025-58897288-5820 传真：025-58897288-5815 电子邮箱：order@genscript.com.cn 网址：www.genscript.com.cn
