# Supplementary material for: Recombinant Plasminogen Activator of the Sandworm (Perinereis aibuhitensis) Expression in Escherichia coli
Source: Bioengineering (Basel). 2024 Oct 15;11(10):1030. doi: 10.3390/bioengineering11101030 (PMC11504054; doi:10.3390/bioengineering11101030)

Certificate of Analysis

Project ID: C5726241G0-2

Construct Information:

Gene Name: pET28-MBP-TEV-pwPlasmin83_PDS418

Clone ID: KK258154

Gene Length: 264 bp

Cloning Vector: PDS418

Cloning Strategy: BamHI/XhoI

Competent cell: TOP10

Growth Temperature: 37 °C

QC Items	Specifications	Results	
**Appearance**	Colorless, clear, free of precipitate or foreign particles	Pass	Clear, colorless, no visible particulates
**Sequence Accuracy**	Sequencing verification match the order requirements	Pass	Matched
**Restriction Digests**	Expected size bands detected in agarose gel electrophoresis	Pass	Matched<br>Shown in attachment 1
**A260/280**	1.8~2.0	Pass	1.96
**Quantity**	Miniprep: 4 μg	Pass	≥ 4 μg
**Additional Tests**	N/A	N/A	

NOTE

Plasmid Storing at	Bacstab Storing at	Glycerol Stock Storing at
-20°C	4°C	-80°C

Certified by: Jing Yu **Date:** 01/23/2024

As a pioneer and leader in gene synthesis technology, and the largest gene synthesis supplier in the US, GenScript has completed over 600,000 genes synthesis projects for scientists around the world. Since our establishment in 2002, we have built the best-in-class capacity and capability for biological research services encompassing gene synthesis and molecular biology, peptide synthesis, custom antibodies, protein expression, antibody and protein engineering, and in vitro and in vivo pharmacology – all with the goal to Make Research Easy.

江苏省南京市江宁科学园雍熙路28号
电话：400-025-8686 025-58897288-5820 传真：025-58897288-5815 电子邮箱：order@genscript.com.cn 网址：www.genscript.com.cn

Attachment 1

Plasmid Construct Map

The gene was cloned in PDS418 by BamHI/XhoI.

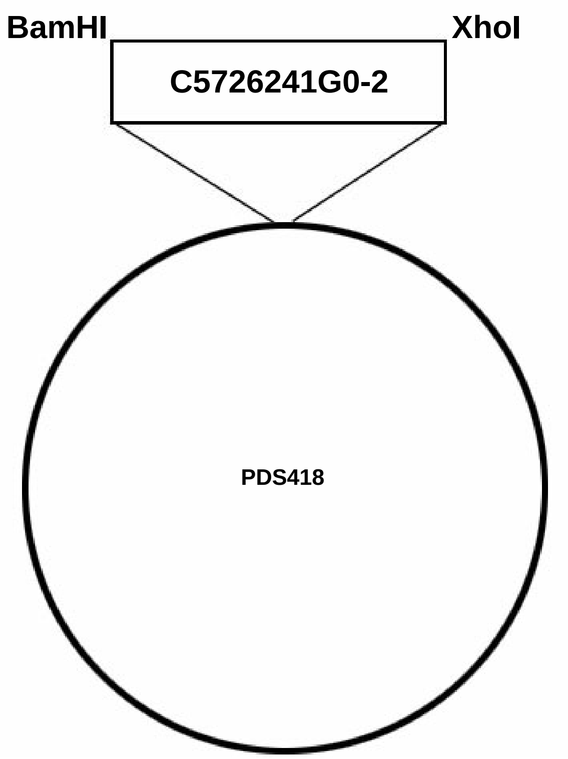

Enzyme Digestion

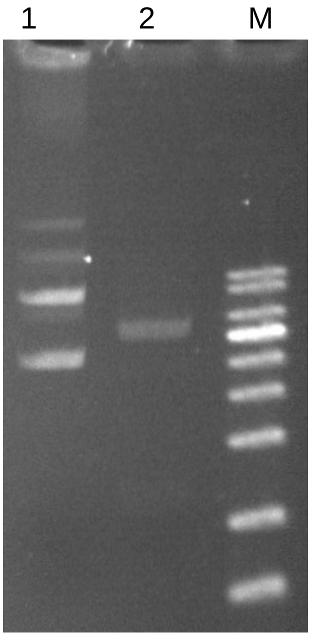

Lane M: KB Ladder
Lane 1:C5726241G0-2 plasmid
Lane 2:C5726241G0-2 plasmid digested
by EcoRI and SmaI

Restriction digest of plasmid DNA:
About 200-1000 ng of plasmid was digested at 37°C for 30-60 minutes and analyzed on 1% Agarose Gel

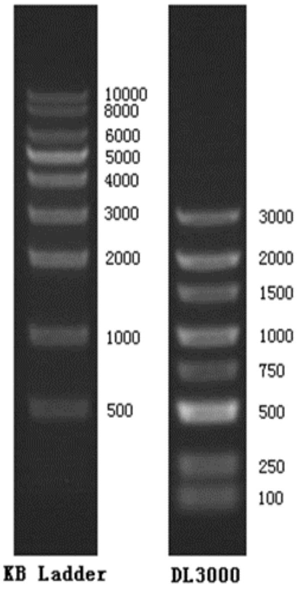

Supplement: Supplementary file 1 [file bioengineering-11-01030-s001.zip › Supplementary Materials S2-CL4329 COA.pdf]
